# Rethinking antimicrobial stewardship paradigms in the context of the gut microbiome

**DOI:** 10.1093/jacamr/dlz015

**Published:** 2019-05-21

**Authors:** Farah Shahi, Kelly Redeker, James Chong

**Affiliations:** 1 Department of Infection, Hull University Teaching Hospitals NHS Trust, Castle Hill Hospital, Cottingham, HU16 5JQ, UK; 2 Department of Biology, University of York, Wentworth Way, Heslington, YO10 5DD, UK

## Abstract

Ongoing concerns over the presence and persistence of antimicrobial resistance (AMR), particularly in Gram-negative bacteria, continue to have significant global health impacts. The gastrointestinal tract, or ‘gut’, environment amplifies AMR in the human gut microbiome, even in the absence of antibiotics. It constitutes a complex and diverse community of organisms, and patterns and alterations within it are increasingly being found to be associated with states of health and disease. Our understanding of the effects of routes of administration of antimicrobials on the gut microbiome is still lacking despite recent advances in metagenomics. In this article we review current evidence for antibiotic effects on gut microbiota and explore possible prescribing and stewardship approaches that would seek to minimize these effects. If we are to preserve existing and new antimicrobials, we need to consider their use in the context of their effect on gut ecology, and the human microbiome in general.

## Introduction

The ongoing emergence of antimicrobial resistance (AMR), particularly in Gram-negative bacteria, is of major global concern. The gastrointestinal tract, or ‘gut’, microbiome is known to be an important ‘amplifier’ of AMR, even in the absence of antibiotics. Patterns and alterations within it are increasingly being found to be associated with states of health and disease. The inability of commonly prescribed antimicrobials to precisely target specific microbial species results in decreased bacterial diversity and load and has the potential to cause overgrowth of resistant bacteria.^1^^,^^2^ Despite recent advances in technology and, with it, a wealth of literature, the concept of the gut microbiome is not a new one. Nord *et al.*^3^ considered the impact of antimicrobial agents on ‘normal’ gut flora, using culture-based methods, in the 1980s; in particular they warned about the use of anti-anaerobe agents resulting in colonization of the gut by Gram-positive and Gram-negative bacteria. Indeed, the use of selective decontamination of the digestive tract (SDD) was already being discussed within the context of maintaining gut ‘ecology,’ limiting new colonization with endogenous and exogenous organisms and preventing sepsis in high-risk patients.^4^ For the purposes of this review article, we will mostly focus on data using emerging technologies, although we acknowledge the ongoing and historical use of culture-based methods to support this.

When antibiotic therapy is required, the current global paradigm favours the use of oral administration, whenever it is possible and safe to do so, with the main goals of: (i) reducing complications of intravenous (iv) access devices, including infections; (ii) avoiding hospital admission and promoting hospital discharge; (iii) reducing economic costs; and (iv) facilitating patient recovery and convenience. To date, the potential differential effects of antibiotic administration route on the gut microbiome have not influenced this paradigm and it remains unclear as to what extent they should.

Zhang *et al.*^2^ challenged the oral paradigm in a mouse model. They found that oral administration of amoxicillin and tetracycline increased the development of AMR genes to a greater extent compared with the same antibiotics administered iv and had differential effects on the gut microbiota. The existing clinical approach of oral administration of antimicrobials whenever possible is undoubtedly robust in most circumstances. However, the potential differential impact of the route of antibiotic administration on the gut microbiome is worthy of consideration with respect to the following: (i) understanding the size and length of this effect for different agents, the clinical consequences thereof, and the antimicrobial prescribing and stewardship implications of such effects; (ii) the importance of developing novel routes of antibiotic administration that are clinically effective, but result in lower gut microbiome exposure;^2^ and (iii) the need for highly specific, very narrow-spectrum (precision) antimicrobials that have less impact on the gut microbiome.

In this article, we discuss how antibiotics affect the gut microbiome and why this is important, and consider the potential roles of different approaches to antimicrobial prescribing and stewardship, and of novel therapeutics in moderating antibiotic-associated adverse effects on the gut.

## The effect of antibiotics on the gut microbiome

Within our gastrointestinal tract there are ∼10^11^–10^12^ microorganisms per gram of content and >1000 different species,^5–7^ with each individual person carrying at least 160 species.^7^ Early attempts at defining the gut microbiome were culture-dependent and only revealed an estimated 10%–30% of the gut microbiota.^8^ PCR techniques and Sanger sequencing furthered our understanding of unculturable organisms.^7^^,^^9^ The study of gut microbial diversity by Eckburg *et al.*^10^ using metagenomic techniques improved our understanding of both the gut microbiome and the potential importance of metagenomic approaches. The six main bacterial phyla found within the gut are Firmicutes, Bacteroidetes, Actinobacteria, Fusobacteria, Proteobacteria and Verrucomicrobia, with the vast majority of gut commensal bacteria being anaerobes.^11^^,^^12^

The development of the gut microbiome is a complex process. Whilst there is a reasonably predictable acquisition of species during early childhood, this may be impacted by timing and route of delivery at birth, breast milk, diet and environment.^13–17^ Early use of antimicrobial agents may have a lasting impact on the developing microbiome and its host, affecting the host’s metabolome and immune system and thereby their health and disease states later in life. Arrieta *et al.*^18^ found that inoculation of germ-free mice with bacteria found to be deficient in children at risk of asthma ameliorated airway inflammation; antibiotic exposure in the first year of life was a significant environmental factor in these children. Cox *et al.*^19^ demonstrated that while low-dose oral penicillin administered to mice in early life may not lead to a prolonged reduction in microbial diversity or abundance, significant differences in adiposity, metabolism and immune function still occur subsequently and are associated with alterations in the characteristics of the bacterial population within the gut microbiome. This suggests that early microbial alterations may continue to affect later human metabolism and health. Gut microbial alterations and their effect on host metabolism and immunity are increasingly associated with non-infection disease states such as inflammatory bowel disease, diabetes, rheumatological conditions, respiratory dysfunction, cancer (where it has been shown to impact the host’s response to chemotherapy), drug metabolism, mental health and recovery after spinal cord injury.^18–25^ Understanding and protecting this microbial community and learning how to manipulate it to provide novel therapeutic strategies and optimize host response to therapy should therefore be of interest to all medical and surgical specialties.

Numerous studies have demonstrated a reduction in microbial diversity and colonization resistance to potential pathogens, such as *Clostridioides* (*Clostridium*) *difficile,* in response to antimicrobial exposure.^1^^,^^6^^,^^16^^,^^26–28^ Culture-based methods demonstrated relatively short-term impacts, finding that ‘normalization’ of the gut microbiota generally occurred within 4 weeks of antibiotic therapy.^3^^,^^5^^,^^27–30^ However, we now have the ability to sequence the remainder of the gut microbiota, which has been challenging to culture to date. Adamsson *et al.*,^31^ even though only using culture-based methods, expressed concern about persistence of resistant microbes after normalization of the gut microbiota following antibiotic exposure. Next-generation sequencing has since confirmed that antibiotics exert longer-term impacts than previously thought. Jernberg *et al.*^6^ and Löfmark *et al.*^32^ described disturbance in the gut *Bacteroides* community, using culture and PCR techniques, which persisted for 2 years after a 7 day course of oral clindamycin (which has notable anti-anaerobe activity). In another study using 454-pyrosequencing, Jakobsson *et al.*^33^ found that the faecal microbiota was affected for up to 4 years following treatment of *Helicobacter pylori* infection; high levels of the macrolide resistance gene *ermB* were also found in some patients.

The gut can therefore act as a reservoir for resistance genes, with many antimicrobials promoting the emergence of resistance through their effects on the gut microbiota.^1^^,^^32^ Importantly, antibiotics selecting for resistant pathogens are not necessarily those the pathogens are nominally or intuitively resistant to; for example, antibiotics other than vancomycin select for the gut expansion of VRE.^34^ Tulstrup *et al.*^35^ recently demonstrated in rats that metronidazole, cefotaxime and vancomycin altered intestinal permeability, although it was unclear as to what extent this affected the microbiota or host health; they also found that amoxicillin and vancomycin initially increase gut bacterial load, presumably owing to expansion of resistant bacteria. Accurately defining the effects of antimicrobials, exactly when effects occur, and how different administration routes impact the gut microbiome using state-of-the-art genomic techniques is therefore of vital clinical and antimicrobial stewardship importance. It is also worth noting that an increase in the expression of AMR genes in the gut microbiota can occur without direct antibiotic exposure, as was shown in a study of 35 Swedish students after travel to the Indian peninsula or Central Africa.^36^ What drove these changes was unclear, but bacterial communities present in the destination environment and changes in diet are likely to have been factors.

## Route of antibiotic administration

Route of administration, antimicrobial metabolism and drug excretion are all thought to be of importance, but our understanding of these, and their clinical and microbiome implications, remains sub-optimal.^1^^,^^28^^,^^30^^,^^37^ Some iv agents are excreted via the biliary system or secreted by the intestine and, unless reabsorbed, can potentially have a greater effect on the gut microbiome than highly bioavailable oral agents that have no or limited biliary or faecal excretion (Table 1).^37–65^ In contrast, oral agents poorly absorbed from the gastrointestinal tract are likely to affect the gut microbiome more than iv agents with low bile or faecal excretion. De Smet *et al.*^66^ recently gave sulfadiazine/trimethoprim to pigs for 5 days by one of three administration routes [oral gavage or via the feed (both twice daily) or intramuscularly by once-daily injection]. Blood, faecal and intestinal tissue samples were taken to assess the concentrations of the two components throughout the bowel. Despite sulphonamides and trimethoprim being highly orally bioavailable and undergoing largely renal excretion, significantly higher concentrations of sulfadiazine were found in the lower gastrointestinal tract than trimethoprim, suggesting unrecognized intestinal secretion of sulfadiazine following absorption. Given that the prescribing of prolonged oral antimicrobial therapy is likely to increase as a result of two recently published clinical trials in orthopaedic infections and endocarditis,^67^^,^^68^ these results may have implications for clinical practice in humans. They highlight our incomplete understanding of the excretion of many drugs, the associated potential gut microbiota effects and the importance of investigating the effects of alternative and novel routes of antibiotic administration on gut microbiota.

**Table 1:** Characteristics of various antibiotics on the WHO Essential Medicines List,^38^ characterized by administration route and CDI risk

Antibiotic	Oral bioavailability/absorption (and excretion)	Excretion (including biliary/faecal) following iv administration	CDI risk (data from NICE evidence summary comprising three meta-analyses^a^)^39^
Amikacin	NA	Renal excretion. Low biliary tract concentrations versus serum.^40^	aminoglycosides: no significant association seen not assessed not assessed
Amoxicillin	∼70% bioavailable.^41^	Predominantly renal. Detected in biliary system following iv administration, but lower concentrations compared with serum.^41^^,^^42^	penicillins: no significant association seen (note subgroup analysis below) penicillins: 5 studies: OR 2.71 (95% CI 1.75–4.21) penicillins: 4 studies: OR 3.25 (95% CI 1.89–5.57)
Amoxicillin/ clavulanic acid	See above.	See above. Clavulanic acid excreted in urine, bile and faeces.^43^	subgroup analysis; penicillin combination antibiotics (e.g. co-amoxiclav and piperacillin/tazobactam): 6 studies: OR 1.54 (95% CI 1.05–2.24) penicillins: 5 studies: OR 2.71 (95% CI 1.75–4.21) penicillins: 4 studies: OR 3.25 (95% CI 1.89–5.57)
Azithromycin	37% plasma bioavailability due to high tissue binding (rather than low GI absorption). Biliary excretion is a major route of elimination.^44^	NA	macrolides: no significant association seen macrolides: 4 studies: OR 2.65 (95% CI 1.92–3.64) macrolides: 3 studies: OR 2.55 (95% CI 1.91–3.39)
Benzylpenicillin	NA	Predominantly renal (60%–90%). Biliary excretion is only a minor fraction.^45^	see amoxicillin see amoxicillin see amoxicillin
Cefalexin	High.^46^	Low biliary excretion; predominantly renal.^40^^,^^46^	subgroup: first-generation cephalosporins, no significant association seen: OR 1.36 (95% CI 0.92–2.00) cephalosporins: 5 studies: OR 5.68 (95% CI 2.12–15.23) (also includes monobactams and carbapenems) cephalosporins: 3 studies: OR 4.47 (95% CI 1.60–12.50)
Cefixime	Low (22%–54%); excreted predominantly unchanged in urine.^47^	NA	subgroup: third-generation cephalosporins, 6 studies: OR 3.20 (95% CI 1.80–5.71) see cefalexin see cefalexin
Cefotaxime	NA	Predominantly renal, but high concentrations in bile.^40^^,^^48^^,^^49^	see cefixime see cefixime see cefixime
Ceftriaxone	NA	High unchanged biliary excretion (∼40%–50%).^40^^,^^50^	see cefixime see cefixime see cefixime
Chloramphenicol	NA	Mostly renal excretion; low active concentrations in bile.^40^	not assessed not assessed not assessed
Ciprofloxacin	70%–80% absolute bioavailability.^51^	Predominantly renal; 15% via faeces after iv (mainly intestinal secretion); 1% bile.^51^^,^^52^	quinolones: 10 studies: OR 1.66 (95% CI 1.17–2.35) quinolones: 5 studies: OR 5.50 (95% CI 4.26–7.11) quinolones: 3 studies: OR 5.65 (95% CI 4.38–7.28)
Clarithromycin	High GI absorption. ∼50% absolute oral bioavailability after 250 mg dose after first-pass metabolism.^53^	20%–40% excreted unchanged in urine (increases with dose increase). 10%–15% in urine as metabolite. Rest is excreted in faeces.^53^	see azithromycin see azithromycin see azithromycin
Clindamycin	High concentrations in bile. 90% plasma binding. Over 90% absorption orally (absolute bioavailability 53% ± 14% for 600 mg dose).^54^	10% of the active drug/metabolites are excreted in the urine, 4% in the faeces; remainder is excreted as inactive metabolites (predominantly faecally).^54^	6 studies: OR 2.86 (95% CI 2.04–4.02) 3 studies: OR 16.80 (95% CI 7.48–37.76) 2 studies: OR 20.43 (95% CI 8.50–49.09)
Doxycycline	93%, almost all absorbed in upper GI tract. Concentrated in bile. 40% of the dose is eliminated in active form in the urine, and 32% in the faeces, after 3 days. High urinary concentrations achieved but in the presence of renal impairment, urinary elimination decreases and faecal elimination increases.^55^	NA	no significant association seen no significant association seen no significant association seen
(Flu)cloxacillin	79% oral absorption.^56^	Predominantly renal. 66% if oral, 76% if parenteral, active drug in urine. Small amount in bile.^56^	see amoxicillin see amoxicillin see amoxicillin
Gentamicin	NA	Renal excretion. Low bile secretion.^40^ Shown to have therapeutic concentrations in bile with once daily dosing.^57^^,^^58^	see amikacin see amikacin see amikacin
Meropenem	NA	Penetrates biliary tract well.^40^^,^^59^ 70% excreted within 12 h unchanged in urine; 2% faecal.^59^	6 studies: OR 1.84 (95% CI 1.26–2.68) see cefalexin: assessed with cephalosporins and monobactams not assessed
Metronidazole	Almost complete oral absorption.^60^	50% in urine in active form and metabolites. 20% in faeces.^61^	not separately assessed not separately assessed not separately assessed
Nitrofurantoin	Rapidly absorbed in upper GI tract but low serum levels.^62^	Urinary excretion.^62^	not separately assessed not separately assessed not separately assessed
Phenoxymethylpenicillin	∼60% absorbed.^63^	Renal excretion. Small amount in bile.^63^	see benzylpenicillin see benzylpenicillin see benzylpenicillin
Piperacillin/tazobactam	NA	High concentrations in bile and 20% biliary excretion.^40^	see amoxicillin/clavulanic acid see amoxicillin/clavulanic acid see amoxicillin/clavulanic acid
Sulfamethoxazole/ trimethoprim	Almost 100% absorption.^65^	Predominantly renal.^64^	5 studies: OR 1.78 (95% CI 1.04–3.05) 4 studies: OR 1.81 (95% CI 1.34–2.43) 3 studies: OR 1.84 (95% CI 1.48–2.29)
Vancomycin	Not usually absorbed in the blood after oral administration (unless bowel wall injury is present, e.g. pseudomembranous colitis).^65^	Parenteral: predominantly renal, with only 5% biliary. Oral: very low in urine. High concentrations in faeces.^65^	not separately assessed not separately assessed not separately assessed

GI, gastrointestinal; NA, not applicable.

a NICE evidence summary of CDI risk with broad-spectrum antibiotics. Compiled data from three meta-analyses: (i) Slimings and Riley (2014)^89^ reviewed hospital-associated CDI; (ii) Brown *et al.* (2013)^90^ reviewed community-associated CDI; and (iii) Deshpande *et al.* (2013)^75^ reviewed community-associated CDI.

## Remaining uncertainties in clinical prescribing

Depending on whether gut microbiota disturbance reaches a maximum point, and if so, when this occurs with different agents, it is likely that shorter courses of antibiotics will impact the gut microbiota less than more prolonged therapy. Spellberg^69^ and Llewelyn *et al.*^70^ recently highlighted the limited evidence supporting the existing antimicrobial-prescribing mantra that a failure to complete a prescribed fixed-length course of antibiotics after clinical resolution increases the risk of either clinical failure and/or AMR. The evidence base for shorter courses of antibiotic therapy for common infections has clearly improved in recent years but remains inadequate; further research is required to define minimum course lengths for common infections of a certain physiological severity that do require antimicrobial therapy, as well as the use of biomarkers to guide their durations. Ultimately, we should be aiming for personalization of antibiotic course lengths in order to minimize gut microbiota exposure and impact whilst optimizing clinical benefit.

Our overall understanding of the human gut microbiome itself, however, still remains limited. The Human Microbiome Project^71^ did not define a ‘healthy’ adult microbiome^17^ and our knowledge of the interplay between bacteria, viruses and fungi within the gut microbiome is poor. It is also recognized that gut antimicrobial exposure may, in certain circumstances, have positive effects through modification of the gut microbiota. SDD is likely to reduce mortality by decreasing gut bacterial overgrowth in critically unwell patients,^4^^,^^72^ but has not been widely implemented, despite a favourable clinical evidence base, because of unproven fears about the potential to promote AMR. The use of amoxicillin or cefdinir has also been shown to improve recovery and decrease mortality in children with severe malnutrition^73^ and rifaximin appears to mediate its beneficial effects in acute or recurrent hepatic encephalopathy via modification of the gut microbiome.^74^ These data suggest that in certain illness states, including those likely to be associated with ‘disturbed’ gut microbiota, the potential adverse effects of antibiotic exposure on the individual and society may be outweighed by clinical gains. Fully understanding the effects on the gut microbiota and beneficial mechanisms of such therapies and the balance between the benefits to the individual versus any potential wider negative ecological impact is clearly of importance.

## Protecting the gut microbiome from antimicrobial damage

### Antimicrobial prescribing and stewardship

Contemplation of the potential antimicrobial stewardship implications of the above suggests the need for a more informed and sophisticated approach to prescribing to optimize gut microbiota protection. For example, apart from prescribing the narrowest-spectrum antimicrobial it is clinically safe to use for the shortest time (a long-accepted stewardship paradigm) it may also be better to: (i) prescribe, and develop, iv agents with limited biliary/faecal excretion (Table 1^38–65^); (ii) use oral agents that limit gastrointestinal tract and gut microbiota exposure; (iii) use antimicrobials without anti-anaerobic effects when clinically possible; or even (iv) stop antibiotic therapy altogether rather than switching from iv to oral when only a small number of further doses are required and clinical response is unlikely to be affected. In the future, there may also be the potential for personalization of drug dosing by therapeutic drug monitoring (with the aim of optimizing efficacy and maximizing suppression of resistance by minimizing drug exposure) through the use of high-fidelity biomarkers of patient recovery as a means of limiting gut antibiotic exposure. For patients in whom these goals are unachievable, mitigation by gut microbiome protection using yet-to-be-developed therapeutics, such as next-generation faecal microbiota transplantation (FMT) or probiotics, may be warranted.

### Microbiome protection therapies

Both FMT and probiotics have shown promise as gut microbiome therapies to mitigate the negative effects of antibiotic exposure. *C. difficile* infection (CDI) has long been known to be associated with antimicrobial prescribing.^75^ Despite first being used in 1958, and with subsequent reports suggesting an ∼90% cure rate in recurrent CDI,^76^ the first randomized controlled trial of FMT was only reported by van Nood *et al*. in 2013.^77^ This trial was relatively small but was stopped early owing to the marked benefits of FMT versus oral vancomycin. Despite this, FMT is yet to be widely implemented in clinical practice, possibly owing to the considerable challenges of use and because the full risks and benefits continue to be debated and investigated. Interestingly, Staley *et al.*^78^ recently found that bacteria associated with secondary bile acid metabolism provide the gut with resistance to CDI, again highlighting the potential importance of protecting the commensal bacteria of the small intestine. The role, if any, of pre- or post-exposure FMT as primary prophylaxis is currently unclear, but it may have potential once the residual concerns about FMT have been resolved and it has evolved into a more easily administered therapy (e.g. in oral pill form).

One recent systematic review found that administration of probiotics within 2 days of the first dose of antibiotic treatment reduced the risk of CDI by >50% (from 3.9% to 1.6%) and early commencement of probiotics at the time of starting antibiotics was likely to be important. There was no significant difference found between the doses, species or formulations studied.^79^ The potential impact of such approaches on mitigating the risk of a patient becoming colonized or infected with antimicrobial-resistant bacteria, or suffering other non-infection health problems, is also unclear, but is theoretically attractive. The development of rapid, inexpensive tests that can quickly identify possible health-impacting disturbance of the gut microbiota may be of importance in future clinical practice. Such tests would identify at-risk patients exposed to a range of interventions (not necessarily just antibiotics) and enable physicians to cost-effectively target microbiome protective therapies to those most likely to benefit.

A different, but elegant approach is the chemical trapping of vancomycin in the gut, which has been found to prevent the selection of VRE *in vitro*.^80^ The use of oral β-lactamases, administered alongside iv β-lactam antibiotics in order to assist the breakdown of antibiotic(s) entering the gut (thereby preserving the microbiome and preventing CDI and other negative effects), is also being investigated: it appears to be an attractive strategy for current and future iv β-lactam agents with microbiota-impacting biliary or faecal drug excretion.^81^ In the future, such considerations and the effects of new agents on the health of the gut microbiome should be incorporated, investigated and publicly reported as part of antimicrobial drug development.^82^

### Avoiding the gut microbiome in treatment

Topical preparations, and local administration, of antibiotics have long been used to treat acute and chronic skin, ear and eye infections, and have been used prophylactically to reduce the risk of surgical site infection (SSI).^83^ Whilst topical or local administration is accepted clinical practice in certain situations (e.g. gentamicin within cement in elective primary joint replacement), use in other clinical scenarios, for example to prevent SSI after colonic surgery or in the treatment of orthopaedic infections, remains controversial. This is often owing to concerns regarding efficacy and the potential for topical agents to encourage AMR or cause local adverse effects, such as rash or itching.^84^ There is therefore a need to synthesize published data and perform further research to identify niches in clinical practice where topical or local administration of antibiotics can reduce or avoid the need for systemic therapy and subsequent gut exposure.

Related to topical and local use of antimicrobials is transdermal administration using dissolvable microneedle patches. Proof-of-principle studies have shown that antibiotics can be successfully administered via this route in mice, leading to systemic concentrations above the MIC of potential target bacteria.^85^ For this route of administration to reduce gut microbiome exposure, however, antibiotics that have low or no biliary or faecal excretion will need to be utilized.

Nebulized administration also has the potential to limit gut antimicrobial exposure, particularly in patients with chronic lung conditions such as cystic fibrosis, a cohort often exposed to systemic therapy. Wenzler *et al.*^86^ highlighted, however, the lack of comparative data and controlled trials in this area. As with transdermal administration, there is also the potential for this route to be used for the treatment of non-lung infections, although pharmacological and drug-delivery challenges remain.^87^ There are also few data on the relationships between the human lung and gut microbiomes and how the latter may be altered by inhaled antimicrobial administration, either by direct ‘leaking’ into the oesophagus during exposure or via biliary excretion of any agent subsequently entering the systemic circulation.

### Use of precision, very-narrow-spectrum agents

The traditional approach to the development of systemic antimicrobial agents has been to develop broad-spectrum ‘blockbuster’ agents that have activity against a wide range of pathogens, but also have considerable ecological adverse effects on human and environmental flora. From a microbiome and AMR perspective, the development of very-narrow-spectrum agents that target a limited number of pathogens (or only one), and therefore impact the human microbiome less, is highly attractive; however, this approach is unlikely to lead to an adequate return on investment for developers within the existing reimbursement framework. Nevertheless, some agents, such as fidaxomicin for CDI, have been brought to market successfully or are in development.^88^ The key to using such drugs cost-effectively, however, will be the development of highly accurate, rapid diagnostics that can be deployed at the bedside or in the clinic.

## Conclusions

Although culture and metagenomic methods have considerably improved our knowledge regarding the effects of antimicrobials on the gut microbiota and the associated risk of AMR gene expression, further research is clearly required. If we are to preserve existing and new antimicrobials, we need to consider their use in the context of their effect on gut ecology, and the human microbiome in general. We also need to challenge existing prescribing paradigms by establishing the clinical evidence base for alternative approaches, such as short-course therapy, stopping iv therapy without an oral switch and alternative routes of administration. The gut microbiome, and approaches to mitigating any negative impact, should be considered during antimicrobial drug development.
